# Social determinants of health and cancer screening implementation and outcomes in the USA: a systematic review protocol

**DOI:** 10.1186/s13643-022-01995-4

**Published:** 2022-06-08

**Authors:** Ariella R. Korn, Callie Walsh-Bailey, Meagan Pilar, Brittney Sandler, Prema Bhattacharjee, W. Todd Moore, Ross C. Brownson, Karen M. Emmons, April Y. Oh

**Affiliations:** 1 Cancer Prevention Fellowship Program, Implementation Science, Division of Cancer Control and Population Sciences, National Cancer Institute, Rockville, MD 20850 USA; 2 Prevention Research Center, Brown School at Washington University in St. Louis, St. Louis, MO USA; 3 Division of Infectious Diseases, Washington University School of Medicine, St. Louis, MO USA; 4 Implementation Science, Division of Cancer Control and Population Sciences, National Cancer Institute, Rockville, MD USA; 5 University of Kansas Medical Center, Kansas, KS USA; 6 Department of Surgery, Division of Public Health Sciences, Alvin J. Siteman Cancer Center, Washington University School of Medicine, Washington University in St. Louis, St. Louis, MT USA; 7 Department of Social and Behavioral Sciences, Harvard T.H. Chan School of Public Health, Boston, MA USA

**Keywords:** Social determinants of health, Cancer screening, Breast cancer, Cervical cancer, Colorectal cancer, Lung cancer, Implementation science, United States

## Abstract

**Background:**

Improving the delivery, uptake, and implementation of cancer screening to meet evidence-based recommendations is needed to reduce persistent cancer health disparities in the USA. Current national public health targets emphasize the role of social determinants of health (SDOH) on cancer screening. However, there remains a need to explicate these linkages, toward the goal of identifying and implementing effective interventions that target and address SDOH to reduce inequities in cancer screening.

**Methods:**

We will conduct a systematic review of English language peer-reviewed original research articles published between 2010 and 2021 that describe observational (qualitative and quantitative) and intervention studies conducted in the USA. In alignment with Healthy People 2030, we will include studies of breast, cervical, colorectal, and/or lung cancer screening. Guided by multiple SDOH frameworks, we will broadly define SDOH by five domain areas: economic stability, education access and quality, healthcare access and quality, neighborhood and built environment, and social and community context. Following systematic literature searches in five databases (Ovid MEDLINE, Embase, CINAHL, Web of Science, Cochrane Library) and piloting of screening procedures, reviewers will independently screen titles/abstracts for potential relevance. Reviewer pairs will then screen full text articles for eligibility criteria. We will extract data items from included articles, including study characteristics, cancer screening intervention information, and coding of SDOH constructs. We will assess study quality using the Mixed Methods Appraisal Tool and synthesize our findings using narrative, descriptive statistics, tables, and figures. Our approach will adhere to the Preferred Reporting Items for Systematic Reviews and Meta-Analysis (PRISMA) recommendations.

**Discussion:**

By completing this systematic review, we will summarize recent literature on SDOH and cancer screening, identify research gaps for inclusion of SDOH, and propose future opportunities for advancing equity in cancer screening by integrating SDOH as part of the implementation context to promote uptake, sustainability, and scale-up in the implementation of screening guidelines.

**Systematic review registration:**

PROSPERO CRD42021276582.

**Supplementary Information:**

The online version contains supplementary material available at 10.1186/s13643-022-01995-4.

## Background

The cancer burden is high in the USA, with approximately 1.9 million new cancer cases and 609,000 cancer deaths estimated in 2022 [1]. Cancer screening can reduce the burden of disease (morbidity and mortality) by early detection of malignancy before the onset of symptoms. Despite evidence-based cancer screening recommendations from the US Preventive Services Task Force [2–5] and the Community Preventive Services Task Force [6–11], cancer screening rates in the USA remain below national goals, and there are well-documented disparities by race, ethnicity, household income, healthcare coverage, and other population groups [12]. Improving the delivery, uptake, and implementation of cancer screening to meet evidence-based recommendations is an important strategy to reduce persistent cancer health disparities in the USA [13].

There is broad recognition that social determinants of health (SDOH) are critical to outcomes across the cancer prevention and control continuum [14, 15]. SDOH are “the conditions in the environments in which people live, learn, work, play, worship, and age” [16] and, in alignment with the US Healthy People 2030 framework, are commonly categorized by five domain areas: economic stability, education access and quality, healthcare access and quality, neighborhood and built environment, and social and community context [17]. Despite interest in SDOH and cancer-related outcomes, there are limited systematic reviews that synthesize evidence across studies of breast, cervical, colorectal, and lung cancer screening—each of which is prioritized in the Healthy People 2030 cancer prevention objectives [18] —with an explicit focus on SDOH. Findings from a 2020 systematic review of 30 economic evaluations of breast, cervical, and colorectal cancer screening suggest that intervening on SDOH is cost-effective for populations who are underserved by screening services and experience disproportionate cancer burden in the USA [19]. Expanding this review of economic evidence, there remains a need to further explicate and summarize the link between SDOH and cancer screening, toward the goal of identifying and implementing effective interventions that consider SDOH to reduce cancer screening inequities [20].

To address this need, we will conduct a systematic review to assess the literature on SDOH and cancer screening, identify research gaps for inclusion of SDOH, and propose future opportunities for advancing equity in cancer screening by integrating SDOH as part of the implementation context to promote uptake, sustainability, and scale-up in the implementation of screening guidelines.

## Methods

The objectives of our systematic review are as follows: (a) summarize qualitative and quantitative findings on the relationships between SDOH and breast, cervical, colorectal, and lung cancer screening in the USA; (b) identify how SDOH have been considered in the implementation of cancer screening interventions; and (c) summarize research gaps and propose opportunities for how SDOH can inform the development of implementation strategies to advance equity in cancer screening. This systematic review will follow recommendations for reporting per the Preferred Reporting Items for Systematic Reviews and Meta-Analysis Protocols (PRISMA-P) [21] and has been registered prospectively with the PROSPERO register of systematic reviews (CRD42021276582). The PRISMA-P checklist is available in Additional file 1. Amendments to the systematic review protocol, if applicable, will be documented on our PROSPERO registration page.

### Eligibility criteria

#### Inclusion criteria

In alignment with the Healthy People 2030 cancer prevention objectives, we will include studies focused on screening for breast, cervical, colorectal, and/or lung cancers [18]. Eligible study outcomes will include measures of cancer screening behavior (e.g., receipt of screening, screening rates) or individual-level factors hypothesized as leading to screening behavior (e.g., knowledge, attitudes, awareness, beliefs). Cancer screening studies must also focus on at least one SDOH within the five domains defined by the Healthy People 2030: economic stability, education access and quality, healthcare access and quality, neighborhood and built environment, and social and community context [17]. Table 1 reports eligibility criteria and examples for SDOH variables by study design type. We will include observational (qualitative and quantitative; independent variable not assigned) and intervention study designs, including pilot and feasibility studies, to achieve our broad study objectives. Table 2 describes the SDOH constructs within the Healthy People 2030 domains, which have been identified and arranged based on established frameworks and definitions from the Healthy People [17], the Kaiser Family Foundation [22], the National Institutes of Health PhenX Toolkit [23], relevant literature [19], and expert input.

**Table 1:** Eligibility criteria and examples for SDOH variables by study design

Study design	Inclusion criteria	Exclusion criteria	Hypothetical examples of eligible studies
Observational, qualitative	Studies that conceptually explore perspectives on how SDOH influence or relate to cancer screening (independent variable not assigned)	Studies that do not conceptually explore perspectives on how SDOH influence or relate to cancer screening	• Focus groups with Chinese-American women to explore **transportation** barriers for cervical cancer screening • Interviews with primary care providers to explore how **health literacy** influences breast cancer screening among female patients
Observational, quantitative	Studies that examine SDOH as the independent variable(s), exposure(s), predictor(s), determinant(s), etc. in relation to cancer screening (independent variable not assigned)	Studies that report SDOH only as demographic or control variable(s)	• Cross-sectional survey to examine the association between **racial/ethnic residential segregation** and lung cancer screening • Prospective cohort study to examine the longitudinal association between **housing instability** and colorectal cancer screening
Intervention	Studies with SDOH as the intervention target(s) or as intervention component(s); studies that analyze SDOH as barriers, facilitators, and/or moderators of intervention effects	Studies that report SDOH only as demographic or control variable(s)	• Non-randomized controlled **social support** intervention to examine changes in breast cancer screening • Secondary analysis of a randomized controlled multicomponent intervention to examine **community educational attainment** as an effect moderator of cervical cancer screening

SDOH in hypothetical examples are bolded.

**Table 2:** SDOH domains and constructs

Economic stability	Education access and quality	Healthcare access and quality	Neighborhood and built environment	Social and community context
• Debt • Employment • Expenses • Food insecurity • Housing instability • Income • Income support (e.g., Supplemental Nutrition Assistance Program (SNAP)) • Medical bills • Poverty and concentrated poverty	• Community educational attainment • Early childhood education • High school graduation • Higher education • Language and literacy skills • Vocational training	• Access to primary care • Affordability • Cost • Financial toxicity of healthcare treatments • Geographical access, proximity, and catchment area • Health insurance coverage • Health literacy • Health policy • Provider availability • Provider linguistic and cultural competency • Quality of care • Telehealth, telemedicine, and mobile health	• Access to healthy foods to support healthy eating, food swamps, and food deserts • Broadband, Internet, and Wi-Fi access • Census tract • Environmental conditions (e.g., air or water quality) • Housing quality and pest infestation • Parks and playgrounds • Safety • Transportation • Walkability	• Adverse childhood experiences • Bias • Civic engagement and participation • Community engagement • Discrimination^a^ • Exposure to violence and trauma • Incarceration and criminal justice system • Racial and ethnic residential segregation • Racism^a^ • Sense of community • Social capital and networks • Social cohesion and integration • Social isolation • Social support and support systems • Social vulnerability • Trust

SDOH constructs were identified and arranged based on established frameworks and definitions from Healthy People [17], the Kaiser Family Foundation [22], the National Institutes of Health PhenX Toolkit [23], relevant literature [19], and expert input. ^a^In the Kaiser Family Foundation’s “Social and Economic Factors Drive Health Outcomes” model [22], discrimination and racism are positioned as underlying constructs that impact all SDOH domains

Eligible publication types will include original research articles published in peer-reviewed journals. Articles must be written in English, include adults aged 18+ years in the USA, and published between 2010 and 2021. The publication range was selected to align with the 2010 passing of the Patient Protection and Affordable Care Act that eliminated cost sharing for cancer screening among other preventive care services, in addition to the 2010 launch of the Healthy People 2020 that introduced an emphasis on SDOH [24].

#### Exclusion criteria

We will exclude records published before 2010 and those in a language other than English. Studies conducted only outside of the USA are ineligible given the unique context of the USA insurance and healthcare provision system, although we will include studies conducted in multiple countries inclusive of the USA if results are reported by country. We will also exclude articles that are not published in a peer-reviewed journal, are nonempirical (e.g., editorials, commentaries), conference abstracts, or missing full text records. We will exclude narrative, scoping, systematic, and other reviews (including meta-analyses); however, we will hand-search citations from relevant reviews to determine if they contain any studies in scope for inclusion for our review. While peer-reviewed study protocols are ineligible, we will “forward search” for eligible articles with study outcomes. We will exclude national surveillance studies that report on cancer screening test receipt or screening rates (e.g., the Centers for Disease Control and Prevention’s *Morbidity and Mortality Weekly Reports* [12]), as these data are well-documented in extant reports and monitored by Healthy People in meeting the cancer prevention objectives [13, 18].

Given the review’s focus on cancer screening for general populations, and following the National Cancer Institute’s Cancer Control Continuum [25], we will exclude studies that focus on the following: primary cancer prevention (e.g., tobacco control, diet, physical activity, and human papillomavirus (HPV) vaccination); genetic testing or screening (sometimes referred to as “cascade screening”) based on family cancer history or among individuals at risk for hereditary cancers; follow-up to abnormal cancer screening results; cancer diagnosis; cancer treatment; and/or cancer survivorship. For studies among cancer survivors, we will exclude articles focused on surveillance screening following a cancer diagnosis (e.g., follow-up mammography) and screening for secondary cancers following a primary cancer diagnosis. Finally, studies that include SDOH variables only as demographic or control variables are ineligible for this review (Table 1).

### Information sources and search strategy

We consulted a research librarian to populate the search terms and develop the search strategy. We performed systematic literature searches in July 2021 in the following five databases: Ovid MEDLINE (US National Library of Medicine); Embase (Elsevier); CINAHL (Cumulative Index to Nursing and Allied Health Literature) Plus (EBSCOhost); Web of Science: Core Collection (Clarivate Analytics); and Cochrane Library: Database of Systematic Reviews (Wiley & Sons). Additional file 2 includes an example search strategy from Ovid MEDLINE. The following limiters were applied during the searches, with some variation by database: English language, publication years 2010–2021, human studies, adult studies, USA studies, and journal articles or review articles (the latter to facilitate reference hand searching). The research librarian entered records obtained in the searches into the EndNote reference management software and performed automated deduplication.

### Selection of sources of evidence

We will use Covidence [26], an online collaborative tool for managing and streamlining reviews, for screening titles/abstracts and full text articles for inclusion and exclusion criteria. For title/abstract screening, we will pilot our screening procedures among our coding team, in which coders will independently apply the screening criteria to a subset of 20 records and then meet to generate consensus. Screening procedures and eligibility criteria will be iteratively refined and applied to additional sets of 20 randomly selected records. Upon reaching satisfactory inter-rater reliability (Fleiss’ kappa ≥ 0.7) [27] and finalizing screening procedures, we will follow a single independent coding approach, in which one coder will independently screen titles/abstracts. We will not code reasons for exclusion in the title/abstract screening phase. The coding team will meet approximately biweekly for inter-rater reliability checks in which all coders will screen the same set of 20 articles; if satisfactory inter-rater reliability is not achieved, the coders will perform additional group coding before resuming independent screening.

For full text screening, we will follow a dual-independent coding approach, in which two coders will independently screen full text articles. Articles must meet all inclusion criteria; if a single exclusion criterion applies to an article, it will be excluded. We will report reasons for exclusion in the full text screening phase in a hierarchical manner, such that the exclusion criterion that appears first in the list of exclusion codes will be applied if there are multiple reasons for exclusion (e.g., if a study is ineligible on publication type and no SDOH construct is assessed, we would apply the ineligible publication type code). If two coders cannot reach consensus on whether to include or the reason for exclusion, a third coder will screen and decide. Prior to full text screening, the coding team will pilot test the protocol with a subset of 10 randomly selected articles. Screening criteria will be iterated, and additional subsets of 10 articles will be coded by the full team until screening procedures are finalized. During the full text screening phase, the coding team will meet approximately biweekly for discussion of articles and eligibility criteria; additional clarification will be added to the screening protocol as needed.

The selection of evidence sources will conclude with three steps: (a) conducting a second pass or “validation check” to ensure that all included articles meet all inclusion criteria, (b) hand searching reference lists of relevant review articles, and (c) forward searching relevant peer-reviewed study protocols for eligible articles that report on study outcomes. When considering additional records in steps (b) and (c), we will follow title/abstract and full text screening procedures outlined above.

### Data extraction

We will develop an instrument for data extraction. Pairs of coders will extract data following a dual nonindependent approach, in which a primary coder will highlight relevant text from included records and enter the data into the instrument, and then a secondary coder will check data entries for completeness and accuracy. Discrepancies will be noted by the second coder and resolved via consensus discussions. If consensus cannot be reached after discussion, a third coder will be consulted. Entered data will not be revised until consensus has been reached. This approach has been applied in other systematic reviews and was chosen because it balances rigor and efficiency [28, 29]. We will pilot test this data extraction approach using at least one full text article for each study design (observational-qualitative, observational-quantitative, intervention) and iteratively adjust the data extraction instrument as needed. Data extracted from full text records will include, but will not be limited to, the following variables (as applicable):

- Article and study information: first author, publication year, study design, study years, and data source(s)
- Study population: age, sex or gender, race/ethnicity, geography, country of origin, and sample size
- SDOH: Healthy People 2030 domains [17] and constructs (Table 2), measures, and level of measurement as informed by the Social Determinants Framework for Cancer Health Equity [14]
- Cancer screening: cancer type(s), screening tests, intervention approach, intervention setting, measures, and outcome(s) in relation to SDOH

### Critical appraisal of individual sources of evidence

Again using a dual nonindependent approach, two coders will apply the Mixed Methods Appraisal Tool (MMAT) to assess study quality [30]. MMAT assesses a wide array of study designs and generates quantitative ratings. Additionally, the MMAT items and extraction template offer a qualitative assessment of study strengths and limitations, from which we will summarize themes and potential opportunities for future research directions or approaches.

### Synthesis of results

We will synthesize results from the systematic review using narrative, tables, and figures to summarize themes and/or quantify characteristics of included articles (e.g., a bar chart showing the number of studies addressing each SDOH domain by cancer screening type). We will group the data by study design (observational-qualitative, observational-quantitative, intervention), and depending on the quantity of articles, also by cancer screening type and SDOH domain. We plan to present the review findings to an expert panel of implementation scientists working in cancer prevention and control to identify (a) potential implications for the development of implementation strategies to advance equity in cancer screening and (b) gaps in research and practice. Expert input will be summarized and reported on alongside other review findings.

## Discussion

With increasing attention to SDOH among funders and researchers working in cancer prevention and control, this systematic review will contribute to a gap in the evidence base by explicating the links between SDOH and cancer screening outcomes and identifying ways that SDOH can inform implementation strategies to advance equity in screening. Strengths of this review are its breadth of included study designs and SDOH constructs, which will allow for a comprehensive examination of the existing literature. Rigor of the systematic review process is supported by use of the PRISMA checklist [21] and collaboration with experts, including a research librarian, to develop the search strategy for five databases. Additionally, our screening and extraction approach balances efficiency with rigor. Using a single coder approach to screen titles/abstracts will allow us to accommodate a high yield of records, whereas pilot testing the screening and data extraction procedures, building in team reliability checks, and data validation will enhance the rigor of our methods in each phase of the review.

Limitations of this systematic review are the exclusion of cancer screening types beyond breast, cervical, colorectal, and lung cancers (e.g., prostate cancer screening), studies among cancer survivors, articles published before 2010, and studies conducted outside of the USA. There is also the potential for publication bias.

We plan to disseminate our review findings via multiple channels (e.g., peer-reviewed journal articles, conference presentations, email listservs, webinars, social media), with the intent to reach scientific and practitioner audiences working in cancer prevention and control. Findings from this systematic review are expected to inform future research and practice that consider SDOH to reduce cancer screening inequities in the USA.

## Supplementary Information

**Additional file 1.** PRISMA-P 2015 checklist.

**Additional file 2.** Example search strategy.

## Data Availability

Data are not reported in this paper. Detailed study procedures will be made available from the corresponding author upon request.

## Abbreviations

MMAT: Mixed Methods Appraisal Tool
PRISMA-P: Preferred Reporting Items for Systematic Reviews and Meta-Analyses Protocols
SDOH: Social determinants of health
