# Supplementary material for: Social determinants of health and cancer screening implementation and outcomes in the USA: a systematic review protocol
Source: Syst Rev. 2022 Jun 8;11:117. doi: 10.1186/s13643-022-01995-4 (PMC9175338; doi:10.1186/s13643-022-01995-4)
Supplement: Supplementary file 2 — Additional file 2. Example search strategy. [file 13643_2022_1995_MOESM2_ESM.docx]

**Additional File 2: Example search strategy**

Database: Ovid Medline (search strategy will be adapted for other databases)

| **Action** | **Term** |
| --- | --- |
| 1 | exp Breast Neoplasms/ or breast cancer.mp |
| 2 | exp Lung Neoplasms/ or lung cancer.mp |
| 3 | exp Colorectal Neoplasms/ or colorectal cancer.mp |
| 4 | exp Uterine Cervical Neoplasms/ or cervical cancer.mp |
| 5 | (Cancer adj2 (breast or colorectal or lung or cervical)).ab,kw,ti. |
| 6 | Or/1-5 |
| 7 | (cancer adj2 (screen* or control or prevention or evaluation or test* or examination or detection or diagnostic)).ab,kw,ti. |
| 8 | (cancer and (disparit* or "health equity" or equit*)).ab,kw,ti. |
| 9 | cancer screening.mp. or exp "Early Detection of Cancer"/ or *Mass Screening/ |
| 10 | Exp Mammography/ or mammography.mp. |
| 11 | (mammogram or "screening mammogram" or "clinical breast exam" or "breast ultrasound").ab,kw,ti. |
| 12 | colonoscopy.mp. or exp Colonoscopy/ |
| 13 | ("FIT test" or "fecal immunochemical test" or "Fecal DNA test" or "FIT DNA" or "multitargted stool DNA test" or "stool test" or " stool DNA test" or "sigmoidoscopy" or "flexible sigmoidoscopy" or "computed tomography colonography" or "CT colonography" or "virtual colonoscopy" or "guaiac based fecal occult blood test" or "gFOBT" or "fecal occult blood test" or "FOBT").ab,kw,ti. |
| 14 | (screen* adj3 (breast or colorectal or cervical or lung)).ab,kw,ti. |
| 15 | screening or "screening disparit*" or "screening adherence").ab,kw,ti. |
| 16 | ("cervical cytology" or "pap test" or "papanicolaou test" or "HPV test" or "human papillomavirus test" or "high risk human papillomavirus test" or "hrHPV" or "cervical cancer cotesting" or "pap co-testing").ab,kw,ti. |
| 17 | ("low dose CT" or "low dose computed tomography" or "LDCT" or "chest radiography" or "sputum cytologic evaluation" or "sputum cytology").ab,kw,ti. |
| 18 | Or/7-17 |
| 19 | 6 AND 18 |
| 20 | exp "Social Determinants of Health"/ or *Social Change/ or *Social Welfare/ or exp Healthcare Disparities/ or exp Health Status Disparities/ or *Residence Characteristics/ or *Sociological Factors/ or exp Health Equity/ |
| 21 | ("social determinant of health" or "social determinants of health" or "sdoh" or "social determining factor*" or "social factor* in health" or "social health determinant*" or "social risk factor" or "social determinant*" or "health determinant*" or "basic need*" or "social need*" or "social services" or "social structural determinant*" or "social injustice").ab,kw,ti. |
| 22 | ((healthcare or health or "cancer health") adj2 (equit* or inequit* or disparit* or equalit* or inequalit*)).ab,kw,ti. |
| 23 | Or/20-22 |
| 24 | exp Social Class/ or *Social Problems/ or exp Socioeconomic Factors/ or *Models, Economic/ or *Economic Status/ or *Unemployment/ or *Employment/ or *Income/ or *Homeless Persons/ or *Housing/ or exp Public Housing/ or exp Public Assistance/ or exp Poverty/ or exp Food Assistance/ or *Food Services/ or *Food Supply/ or *Hunger/ |
| 25 | ("economic stability" OR unemployment OR employment OR "job security" OR "economic opportunit*" OR "low income" or debt or bankruptcy OR "social class*" OR "socioeconomic status" OR "socioeconomic factor*" OR "socioeconomic disadvantage*" OR "socioeconomic inequalit*" OR "economic disparit*" OR "economic disadvantage*" OR "financial debt" OR "financial instability" OR "resource poor" OR "social status" OR "social condition*" OR poverty OR poor OR "lower class" OR "middle class" OR "economic status" OR "economic factor*" OR housing OR lodging* OR domicile* OR "living arrangement*" OR residence* OR dwelling* OR homeless OR homelessness OR "living accommodation*" OR residential OR "rental assistance" OR "rent assistance" OR "public assistance" OR hunger OR hungry OR "food stress" OR "food insecurity" OR "food insecur*" OR "food hardship" OR "food insufficienc*" OR "food assistance" OR "food suppl*" OR "food security*" OR "food aid" OR "supplemental nutrition assistance" OR SNAP OR "food stamp*" OR "WIC program*" OR "women infants and children program" or debt or eviction* or bankruptcy or foreclosure or mortgage or rural*).ab,kw,ti. |
| 26 | Or/ 24-25 |
| 27 | exp Social Environment/ or exp Social Support/ or *Psychosocial Support Systems/ or *Psychosocial Deprivation or *Social Isolation/ or exp Racism/ or exp Prejudice/ or *Social integration/ or exp Violence/ or exp Race Relations/ or * Refugees/ or *working poor/ or exp Social Stigma/ or *social control, informal/ or exp Social networking/ |
| 28 | (social adj2 (context or environment or ecolog* or integrat* or isolat* or trust or vulnerability or cohes* or capital or organi?ation)).ab.kw.ti |
| 29 | (racial adj2 (discriminat* or bias or prejudice or cohes*)).ab,kw,ti |
| 30 | ((community or civic) adj2 (engagement or cooperation or context or cohes* or violence or "sense of")).ab,kw,ti. |
| 31 | (violence adj2 (exposure or experience* or neighbo?hood or witness* or victim)).ab,kw,ti |
| 32 | ("structural racism" or "systemic racism" or "institutional racism" or segregation or "residential segregation" or "ethnic group*").ab,kw,t |
| 33 | Or/27-32 |
| 34 | exp Environmental Exposure/ or *Environment Design/ or exp Built Environment/ or exp Urban renewal/ or exp Parks, Recreational/ or exp Transportation/ |
| 35 | (transportation or commute or "public transit" or highway* or subway or transit or "mixed use" or pedestrian or walkab*).ab,kw,ti |
| 36 | ("green space*" or park$1 or playground* or sidewalk* or "community garden$1" or "food desert" or "grocery store density" or "fast food" or "restaurant density" or "healthy food availability" or HFAI or "nutrition environment measures" or NEMS).ab,kw,ti. |
| 37 | ((owner adj2 (vehicle or auto* or car)) or "alcohol outlet*" or neighbo?rhood or "housing quality" or "urban environment" or "inner cit*").ab,kw,ti |
| 38 | OR/34-37 |
| 39 | exp Educational Status/ or exp Literacy or exp Education/ |
| 40 | (educational* adj2 (level? or status or attainment or achievement or access)).ab,kw,ti. |
| 41 | (litera$2 adj2 (health or cancer or English)).ab,kw,ti. |
| 42 | (degree adj2 (university or college or advanced or "high school")).ab,kw,ti. |
| 43 | (GED or "high school graduate" or "high school dropout" or "high school completion").ab,kw,ti. |
| 44 | Or/ 39-43 |
| 45 | exp Cultural Competency/ or exp “Culturally Competent Care”/ or exp Patient Navigation/ or *acculturation/ or exp Cross-Cultural Comparison/ or exp Cultural Diversity/ or exp Health Services Accessibility/ or *medicare/ or *Medicaid/ or exp Medically Uninsured/ or exp Insurance Coverage/ or exp Insurance, Health/ |
| 46 | ("patient navigat*" or "transcultural care" or "culturally appropriate care" or "culturally competent care" or "cultural care" or "cultural competenc*").ab,kw,ti. |
| 47 | ((healthcare or care or provider or "health services" or insurance) adj2 (access* or availab*)).ab,kw,ti. |
| 48 | Or/45-47 |
| 49 | 23 or 26 or 33 or 38 or 44 or 48 |
| 50 | ("US" or "United States" or "United States America" or America or American or North America).af. |
| 51 | 19 AND 49 |
| 52 | 50 AND 51 |
| 53 | Limit 52 to (english language and humans and yr="2010-2021" and (journal article or meta analysis or "review" or "scientific integrity review" or "systematic review")) |
